# Cerebellar primary central nervous system lymphoma: Case series report

**DOI:** 10.1016/j.ijscr.2022.107440

**Published:** 2022-07-21

**Authors:** Minglian He, Jun Zhong, Xuegang Li, Yujie Chen, Fei Li

**Affiliations:** Department of Neurosurgery and State Key Laboratory of Trauma, Burn and Combined Injury, Southwest Hospital, Third Military Medical University (Army Medical University), Chongqing 400038, China; Chongqing Clinical Research Center for Neurosurgery, Southwest Hospital, Third Military Medical University (Army Medical University), Chongqing 400038, China; Chongqing Key Laboratory of Precision Neuromedicine and Neuroregenaration, Southwest Hospital, Third Military Medical University (Army Medical University), Chongqing 400038, China

**Keywords:** Primary central nervous system lymphoma, Cerebellum, Diffuse large B-cell lymphoma, Case series

## Abstract

**Introduction and importance:**

Primary central nervous system lymphoma (PCNSL) is a rare cranial malignant haematological tumour. PCNSL in the cerebellar region is less common than PCNSL in other encephalic regions. A diagnosis of cerebellar PCNSL is relatively difficult to make due to its diverse imaging manifestations. The aim of this case series report is to determine whether surgery could be used to confirm the diagnosis of cerebellar PCNSL and the effect of surgical treatment.

**Methods:**

We report 3 cases of cerebellar PCNSL that underwent neuronavigation microsurgery under general anaesthesia. The operation was performed by author 3 and author 5. One patient underwent left lateral ventricular drainage on the fourth and tenth days after the operation due to postoperative obstructive hydrocephalus. All patients received chemotherapy or radiotherapy after histological confirmation.

**Outcomes:**

All patients' tumours were completely removed. One patient developed obstructive hydrocephalus twice during the perioperative period after the operation, was given drainage, and then recovered from the hospital. The other two patients recovered and were discharged smoothly without complications. One patient died 9 months after the operation, and the other two patients survived. The prognosis of 3 patients was related to tumour size and timely follow-up chemo-radiation therapy.

**Conclusion:**

The histology of all patients showed diffuse large B-cell lymphoma (GCB phenotype). Suspicious cerebellar PCNSL patients should undergo surgery to confirm the diagnosis, followed by radiotherapy and chemotherapy.

## Highlights

• Primary central nervous system lymphoma is rarely discovered in the cerebellum.
• A diagnosis of cerebellar PCNSL is relatively difficult to make.
• The age of disease onset was relatively young in our cases.
• Cerebellar PCNSL patients should undergo surgery to confirm the diagnosis.

## 1 Introduction

Primary central nervous system lymphoma (PCNSL) is a rare and aggressive malignant primary central nervous system disease that constitutes approximately 3 % of non-Hodgkin lymphoma (NHL) and 2.4–3 % of all brain tumours [1], [2]. Histologically, most PCNSL cases are diffuse large B-cell lymphoma; thus, this tumour is extremely aggressive and carries a poor prognosis. PCNSL can invade the parenchyma but remains confined within the central nervous system. Brain hemispheres are the most common lesion site, and most of them are localized in the deep frontal lobe. The callosum can also be invaded. However, PCNSL is rarely discovered in the cerebellar region; only approximately 9 % of all PCNSLs are located in this region [3].

PCNSL lacks specific manifestations and is easily misdiagnosed clinically. The diagnosis of this disease requires pathological biopsy and immunohistochemical results. Therefore, the diagnosis of the disease is difficult. The choice of salvage treatment depends on a patient's age, previous treatment and response, performance status, and comorbidities at the time of relapse. The role of surgery in PCNSL is limited, but it may be considered under special circumstances, for example, if there is evidence of increased intracranial pressure from a large lesion with acute symptoms of brain herniation [4]. Herein, we describe 3 cases of PCNSL involving the cerebellum. The diagnosis was made clearly after the operation, and good results were achieved. This study provides a reference for the diagnosis and treatment of this kind of disease.

## 2 Case series

### 2.1 Case 1

**Fig. 1:**
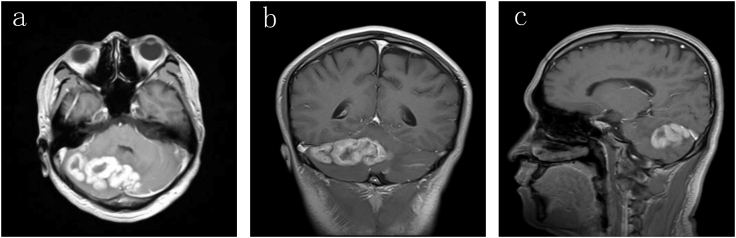
Magnetic resonance imaging showing cerebellar mass lesions within the bilateral cerebellar hemispheres with strong enhancement.

A 22-year-old man (junior high school) presented with dizziness and headache for 1 month, aggravated and accompanied by nausea and vomiting for 1 week. He had no surgical, drug, or other relevant medical history. He smoked 20 cigarettes/day for the past 7 years, drank occasionally, had no history of recreational drug use, and had no allergies or family history. He engaged in transportation. Magnetic resonance imaging (MRI) showed mixed signals in the bilateral cerebellum, mostly on the right side (Fig. 1). The brain stem and fourth ventricle were compressed. Three days after the patient was admitted to the hospital, he presented with severe dizziness, headache, vomiting and vision loss. We immediately performed head CT, and obstructive hydrocephalus was found. We subsequently performed right cerebellar tumour excision under general anaesthesia with a surgical microscope by neuronavigation. The operation lasted 405 min, and complete resection of the tumour was performed under a microscope. The histology of this tumour was non-Hodgkin B-cell lymphoma (DLBCL, diffuse large B-cell lymphoma, GCB phenotype). The patient recovered smoothly without complications. Three weeks after surgery, the patient started receiving 8 rounds of chemotherapy, and the therapeutic strategy was cyclophosphamide (CTX, 1.2 g, 0.8 g/m^2^, day 1) + epirubicin (EPI, 100 mg, 60 mg/m^2^, day 1) + vincristine (VCR, 1.8 mg, 1.2 mg/m^2^, day 1) + prednisone (PDN, 100 mg, po, day 1–5, q3w). The patient did not receive radiotherapy. Unfortunately, PCNSL could not be suppressed by this therapeutic strategy, and the tumour invaded other brain sections. This patient died within 9 months.

### 2.2 Case 2

**Fig. 2:**
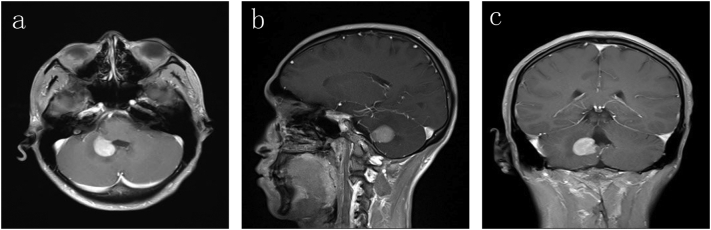
Magnetic resonance imaging showing a high enhancement signal in the right cerebellum.

A 26-year-old unemployed man (junior high school) presented with dizziness and unsteady gait for more than 10 days. He had no surgical, drug, or other relevant medical history. He smoked 20 cigarettes/day for 10 years, drank occasionally, had no history of recreational drug use, and had no allergies or family history. Enhanced MRI revealed a high signal in the right cerebellum (Fig. 2). The patient exhibited cerebellar nervous function damage. After the preoperative examination was performed and no contraindications were found, right cerebellar tumour resection under general anaesthesia with a surgical microscope by neuronavigation was performed to rescue cerebellar neurological function. The operation lasted 497 min, and complete resection of the tumour was performed under a microscope. The patient underwent left lateral ventricular drainage on the fourth and tenth days after the operation due to postoperative obstructive hydrocephalus. The histology of this tumour was non-Hodgkin B-cell lymphoma (DLBCL, diffuse large B-cell lymphoma, GCB phenotype). Molecular determination showed that this PCNSL exhibited the following: CK (−), CD3 (−), CD20 (+), Ki-67 (95 %+), CD10 (+), CD79a (++), C-myc (30 %+), BCL-6 (+), and P53 (+). After surgery, the patient received methotrexate + cytarabine chemotherapy once and no radiotherapy due to intolerance. The patient has survived and is generally in good condition.

### 2.3 Case 3

**Fig. 3:**
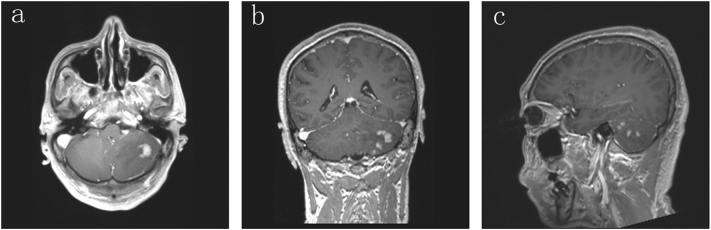
Magnetic resonance imaging enhancement is very evident in the cerebellum (bilateral), with multiple masses observed, and the largest mass is localized in the left cerebellum.

A 54-year-old man (junior college) presented with an occipitalial sore for 1 week. He had no surgical, drug, or other relevant medical history, no smoking or drinking, no history of recreational drug use, and no allergies or family history. He engaged in landscape monitoring. MRI revealed multiple nodular signals with low T1 and slightly high T2 in both cerebellar hemispheres. The FLAIR sequence showed annular hyperintensity, and enhancement was very evident on the enhanced scan (Fig. 3). After the preoperative examination was performed and no contraindications were found, we performed right cerebellar tumour resection under general anaesthesia with a surgical microscope by neuronavigation to explore its histology to guide subsequent therapy. The operation lasted 291 min, and complete resection of the tumour was performed under a microscope. The histology was non-Hodgkin B-cell lymphoma (diffuse large B-cell lymphoma, GCB phenotype). The patient then received 6 rounds of radiotherapy and no chemotherapy. To date, this patient has not experienced tumour progression and is generally in good condition.

## 3 Discussion

Here, we report 3 cases of cerebellar PCNSL that were treated by surgical resection. The average incidence age was 34 years in the three patients, and the age of disease onset was relatively young in our cases. The patients in two cases were younger than 30 years. The operation was performed by author 3 and author 5, and the surgeons are experienced professors. Histopathological diagnosis was made after surgery, and all cases were diffuse large B-cell lymphoma, which was consistent with the histology of most PCNSLs. One patient died at 9 months after diagnosis, and the patient received surgery + chemotherapy (CHOP protocol, 8 rounds). The other two patients are still alive. Of the surviving patients, one patient received surgery + radiotherapy (6 rounds), and the other received surgery + chemotherapy (only once). The different survival times of these 3 patients may be due to the size of the tumour.

The most common aetiology of PCNSL is immunodeficiency, which includes acquired or congenital immunodeficiency [5], [6]. However, the cases that we reported herein had no obvious immunodeficiency, as the leukocytes and lymphocytes of these patients were not abnormal before presentation. PCNSL is a rare malignant tumour, accounting for only approximately 3 % of all cranial tumours. In America, approximately 1600 patients are affected by PCNSL per year. It seems more common in elderly patients than in younger patients [7]. In this report, the three patients were relatively young, with ages of 22, 26, and 54 years. The most common sites of PCNSL are the supratentorial region (approximately 80 %), including the lobe, callosum and basal ganglia. Cerebellar and spinal PCNSLs are rarer than supratentorial PCNSLs, and cerebellar PCNSLs account for only approximately 9 % of all cases [3]. The majority of PCNSLs are composed of diffuse large B cells and express the pan-B-cell markers CD19, CD20, CD22 and CD79a, which are detected in approximately 95 % of cases [8].

Because of the diverse manifestations on CT or MR, PCNSL is not easily diagnosed. Thus, cytology and histology of biopsy- or resection-acquired lesion tissues are needed to diagnose this disease. In addition, the therapeutic strategy of PCNSL mainly includes radiotherapy, chemotherapy and surgery to prolong survival. PCNSL is highly sensitive to radiation; thus, after diagnosis, patients should undergo radiation therapy. Moreover, PCNSL is sensitive to some chemotherapy agents, such as methotrexate, procarbazine, cytarabine and pharmorubicin. Surgery is usually not the first consideration for PCNSL unless the mass effect of the tumour is so severe that it threatens patient survival. Upon diagnosis, the therapeutic strategy usually includes radiotherapy and chemotherapy. However, even with timely therapeutic intervention, the prognosis of PCNSL remains poor. We are accumulating more such cases in the later stage to guide clinical treatment.

## 4 Conclusion

PCNSL is a rare malignant cranial tumour, especially when the lesion site is in the cerebellum. Suspicious cerebellar PCNSL patients should undergo surgery to confirm the diagnosis, followed by radiotherapy and chemotherapy.

## Statement

This work has been reported in line with the SCARE criteria [9].

This work has been reported in line with the SCARE 2020 criteria [10].

This work has been reported in line with the PROCESS Guidelines 2020 (www.processguideline.com) criteria [11].

This study is a retrospective, single-centre, consecutive case series.

The patient management setting is a teaching/district general hospital, and the data collection time frame is December 2021 to July 2022.

## Provenance and peer review

Not commissioned, externally peer-reviewed.

## Sources of funding

This study was supported by the 10.13039/501100001809National Natural Science Foundation of China (81672783).

## Ethical approval

This study was approved by the local ethics committee.

## Consent

Written informed consent was obtained from the patient for publication of this case report and accompanying images. A copy of the written consent is available for review by the Editor-in-Chief of this journal on request.

## Authors' contributions

Ms. Minglian He: The conception and design of the study, analysis and interpretation of data, drafting the article, final approval of the version to be submitted.

Dr. Jun Zhong: Acquisition of data, analysis and interpretation of data, drafting the article, final approval of the version to be submitted.

Dr. Xuegang Li: Acquisition of data, analysis and interpretation of data, final approval of the version to be submitted.

Dr. Yujie Chen: The conception and design of the study, analysis and interpretation of data, critical revision for important intellectual content, final approval of the version to be submitted.

Dr. Fei Li: The conception and design of the study, analysis and interpretation of data, critical revision for important intellectual content, final approval of the version to be submitted.

## Research registration

Not applicable.

## Guarantor

Ms. Minglian He.

Dr. Fei Li.

## Declaration of competing interest

No benefits in any form have been received or will be received from a commercial party related directly or indirectly to the subject of this article.

## References

[1] Schlegel U. (2009). Primary CNS lymphoma. Ther. Adv. Neurol. Disord..

[2] Houillier C., Soussain C., Ghesquières H. (2020). Management and outcome of primary CNS lymphoma in the modern era: an LOC network study. Neurology.

[3] Datta A., Gupta A., Choudhury K.B. (2014). Primary cerebellar B cell lymphoma: a case report. Int. J. Case Rep. Images.

[4] Han C.H., Batchelor T.T. (2017). Diagnosis and management of primary central nervous system lymphoma. Cancer.

[5] Schabet M. (1999). Epidemiology of primary CNS lymphoma. J. Neuro-Oncol..

[6] Bhagavathi S., Wilson J.D. (2008). Primary central nervous system lymphoma. Arch. Pathol. Lab Med..

[7] Ostrom Q.T., Patil N., Cioffi G., Waite K., Kruchko C., Barnholtz-Sloan J.S. (2020). CBTRUS statistical report: primary brain and other central nervous system tumors diagnosed in the United States in 2013–2017. Neuro-Oncology.

[8] Yang X.L., Liu Y.B. (2017). Advances in pathobiology of primary central nervous system lymphoma. Chin. Med. J..

[9] Agha R.A., Borrelli M.R., Farwana R. (2018). The SCARE 2018 statement: updating consensus Surgical CAse REport (SCARE) guidelines. Int. J. Surg..

[10] Agha R.A., Franchi T., Sohrabi C., Mathew G., Kerwan A., SCARE Group (2020). The SCARE 2020 guideline: updating consensus Surgical CAse REport (SCARE) guidelines. Int. J. Surg..

[11] Agha R.A., Sohrabi C., Mathew G. (2020). The PROCESS 2020 guideline: updating consensus Preferred Reporting Of CasESeries in Surgery (PROCESS) guidelines. Int. J. Surg..

